# Resilience Coaching for Adolescent Chronic Musculoskeletal Pain: Protocol for a Pilot Randomized Controlled Trial of Promoting Resilience in Stress Management (PRISM)

**DOI:** 10.2196/73385

**Published:** 2025-07-22

**Authors:** Sabrina Gmuca, Mackenzie McGill, Nellie Butler, Rui Xiao, Peter F Cronholm, Jami F Young, Tonya M Palermo, Pamela F Weiss, Abby R Rosenberg

**Affiliations:** 1 Division of Rheumatology Department of Pediatrics Children's Hospital of Philadelphia Philadelphia, PA United States; 2 Clinical Futures Children's Hospital of Philadelphia Philadelphia, PA United States; 3 PolicyLab Children's Hospital of Philadelphia Philadelphia, PA United States; 4 Perelman School of Medicine University of Pennsylvania Philadelphia, PA United States; 5 Department of Biostatistics, Epidemiology and Informatics Perelman School of Medicine University of Pennsylvania Philadelphia, PA United States; 6 Center for Public Health, Leonard Davis Institute of Health Economics Department of Family Medicine and Community Health University of Pennsylvania Philadelphia, PA United States; 7 Department of Child and Adolescent Psychiatry and Behavioral Sciences Children's Hospital of Philadelphia Philadelphia, PA United States; 8 Department of Anesthesiology and Pain Medicine School of Medicine University of Washington Seattle, WA United States; 9 Center for Child Health, Behavior and Development Seattle Children’s Research Institute Seattle, WA United States; 10 Department of Supportive Oncology Division of Pediatric Palliative Care Dana-Farber Cancer Institute Boston, MA United States; 11 Department of Pediatrics Boston Children's Hospital Boston, MA United States; 12 Department of Pediatrics Harvard Medical School Boston, MA United States

**Keywords:** resilience, chronic pain, chronic musculoskeletal pain, pediatric rheumatology, pilot study

## Abstract

**Background:**

Levels of self-perceived psychological resilience are low to moderate among youth with chronic musculoskeletal pain (CMP). Furthermore, resilience has been associated with symptom severity in CMP. Resilience coaching programs may therefore be of benefit in the nonpharmacologic management of adolescent CMP and may serve as an adjunctive way to access mental health services in an approachable and affordable way.

**Objective:**

The main goal of the study is to assess the feasibility, acceptability, and preliminary efficacy of the resilience coaching program called Promoting Resilience in Stress Management (PRISM) and to obtain the data needed to plan a larger trial.

**Methods:**

The Resilience Coaching for Adolescents with Chronic Musculoskeletal Pain pilot study is an investigator initiated, 2-arm, randomized controlled trial (RCT) of PRISM in the interdisciplinary management of CMP among adolescents. The study will compare usual care versus PRISM+usual care among adolescents newly diagnosed with CMP in the outpatient setting. One caregiver per patient will also be enrolled. The control group will receive usual care with no specific intervention. The treatment arm will receive PRISM, which is a remotely delivered, 1-on-1resilience coaching program, consisting of 4 required skill-based sessions and an optional final session. Sessions will be delivered every 1-2 weeks, lasting about 3 months in total. The primary outcome is the Functional Disability Inventory (FDI) score at 3 months postrandomization. The secondary objectives are to evaluate potential patient- and caregiver-level moderators of PRISM and identify facilitators of and barriers to engagement in PRISM. The estimated sample size is 65 patient-caregiver dyads per group, for a total of 130 dyads.

**Results:**

The trial is currently open. Initial Institutional Review Board approval was obtained on April 4, 2023, and protocol version 4 was amended on January 14, 2025. Recruitment began on May 8, 2023, and recruitment is anticipated to be completed on August 1, 2025.

**Conclusions:**

Resilience coaching has demonstrated excellent feasibility, acceptability, and efficacy in teenagers with chronic illness; however, evidence to support its use in adolescent CMP is lacking. Resilience coaching has the potential to improve patient outcomes in this population. This pilot RCT will demonstrate acceptability, feasibility, and preliminary efficacy and reveal critical barriers to and facilitators of engagement. This will inform a larger multisite trial to evaluate the definitive efficacy of the intervention.

**Trial Registration:**

ClinicalTrials.gov NCT05834725; https://clinicaltrials.gov/study/NCT05834725.

**International Registered Report Identifier (IRRID):**

DERR1-10.2196/73385

## Introduction

Chronic musculoskeletal pain (CMP), or pain in the bones, joints, and tissues lasting longer than 3 months [1], is a common condition affecting up to 25% of children and adolescents [2]. Many adolescents with CMP experience significant psychological distress [3], exhibiting higher levels of depression and anxiety than youth without CMP [4]. Further elucidating the relationship between stress and pain, higher pain intensity in adolescents with CMP is associated with greater emotional and behavioral challenges [5]. Along with these mental health challenges, CMP is also associated with functional disability in various domains, including school attendance, reduced social contact, sleep problems, and family functioning [6].

The current standard of care treatment for adolescent CMP consists of nonpharmacological, interdisciplinary care combining physical, occupational, and psychological therapies [7-10]. These treatments require intensive physical activity, which can be both physically and emotionally taxing. Mental health interventions are a key element of this multimodal treatment approach, with cognitive behavioral therapy (CBT) being the most common [10]. However, many patients encounter months-long waitlists to access specialized interdisciplinary pain care.[11]

Considering that experiencing chronic pain and adhering to CMP treatment recommendations can be significant life stressors, researchers have proposed psychological resilience as a method of coping with chronic pain [12,13]. Adolescents with CMP have been demonstrated to have low-to-moderate self-perceived psychological resilience [14], which is defined as “a dynamic process of positive adaptation or continued development in the context of significant adversity” [15,16]. Higher levels of resilience in youth with CMP have been found to correlate with lower symptom severity, increased function [14], and reduced odds of suicidality [17]. Thus, further research is needed to establish whether interventions targeted at increasing resilience can improve pain coping, ultimately leading to improvements in pain-related clinical outcomes, such as functional disability and psychological distress. Brief resilience coaching programs also have the potential to alleviate difficulties in accessing specialized pain care, as coaching can be readily initiated in the time between initial diagnosis and a patient’s first specialized pain clinic appointment.

Promoting Resilience in Stress Management (PRISM) is a 1-on-1 resilience coaching program consisting of 5 skill-based sessions offered in person or remotely via videoconferencing or telephone for adolescents with chronic illness. PRISM’s efficacy has been shown via improved resilience, health-related quality of life, and mental health outcomes for adolescents and young adults with serious illnesses, including cancer, type I diabetes, and cystic fibrosis [18-21]. Although CBT and other evidence-based psychological strategies have strong evidence supporting their use in the treatment of pediatric chronic pain [22], the use of a resilience framework and resilience coaching has not been formally examined among youth with CMP.

Our prior single-arm pilot study to assess PRISM’s feasibility and acceptability among youth with CMP was the first effort to investigate resilience coaching in this patient population. In this study, participants completed patient-reported outcome measures (PROMs), pre- and postintervention, and both patients and caregivers provided qualitative feedback. The feedback was highly positive, reinforcing our hypothesis that PRISM offers an acceptable alternative psychosocial intervention for youth who either struggle to access mental health services or face stigma associated with them. The study also confirmed that PRISM is feasible and acceptable among youth with CMP, while exploratory analyses suggested improvements in resilience, functional disability, and psychological distress [23]. Significant changes were not observed in pain intensity, pain catastrophizing, and global health; however, we did not have a control group for comparison, nor were we powered to examine efficacy. Based on the findings of this pilot study, we determined that PRISM holds promise for the treatment of adolescent CMP and that additional work is needed to evaluate the efficacy of PRISM compared to usual care and explore factors informing its implementation.

One challenge observed during the single-arm pilot study was that 55% of participants were already engaging in psychological treatment, limiting our ability to attribute changes in PROMs solely to the effects of PRISM. Qualitative interview findings also suggested that the intervention could be of more value to those who were naive to psychological counseling. We determined that future research is needed to separate the effects of PRISM from those of CBT by excluding patients who are actively receiving CBT at the time of screening. We also intended to limit future studies to patients with mild or greater impairment due to pain in order to direct the intervention toward adolescents most in need, while increasing the possibility of demonstrating an improvement in functional disability scores. Lastly, to further assess the durability of PRISM, we planned to extend data collection beyond 3 months to include a 9-month follow-up survey. We also aimed to incorporate participant feedback from our single-arm pilot trial regarding overall helpfulness and benefits, reasons for deciding to participate, session logistics, accessibility, mental health perceptions, and caregiver interest in resilience into future protocols to better meet the needs of patients.

In this paper, we present the protocol for an ongoing pilot phase 2 randomized controlled trial (RCT) of PRISM for adolescents with CMP. The goal of this study is to determine whether the addition of PRISM to usual care demonstrates greater improvements in functional disability than usual care alone. Usual care was chosen as the comparator group to best mimic the clinical real-world setting and because denial of these services would not be ethically sound. Secondary aims include (1) determining the impact of PRISM on pain intensity and psychological distress, (2) exploring moderators of intervention effects, and (3) examining implementation outcomes of, facilitators of, and barriers to intervention engagement. This work provides the basis for a future definitive multicenter RCT to evaluate the efficacy of the intervention and feasibility of delivering resilience coaching across sites, as this will facilitate and support implementation of resilience coaching within usual care for CMP.

## Methods

### Study Design and Setting

The study was registered at ClinicalTrials.gov (NCT05834725) on April 28, 2023. This study is a single-center pilot, 2-arm, RCT using a mixed methods approach (quantitative patient outcome measures combined with qualitative interview data). Participants are recruited from an outpatient pediatric rheumatology clinic and an interdisciplinary pain clinic at the Children’s Hospital of Philadelphia (CHOP) in Philadelphia, PA, USA. See Multimedia Appendix 1 for the SPIRIT (Standard Protocol Items: Recommendations for Interventional Trials) checklist.

### The PRISM Intervention

**Figure 1:**
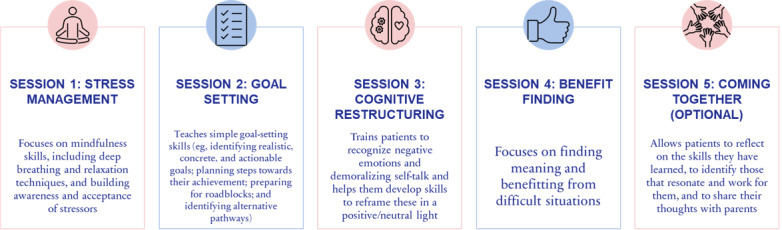
Overview of sessions in the PRISM program. PRISM: Promoting Resilience in Stress Management.

PRISM consists of 4 required skill-based sessions that focus on stress management, goal setting, cognitive restructuring, and benefit finding (see Figure 1). The optional fifth “coming together” session focuses on discussing with a family member, with the participant’s permission, the skills that the participant found beneficial, the ones they did not, and how they can continue to implement the skills learned in the future once the program finishes. Each session is around 30-50 minutes, with sessions scheduled 1-2 weeks apart. Participants are given access to “cheat sheets” and worksheets for each session and a mobile app that can be used to practice the skills learned.

PRISM coaches have a bachelor’s degree (at minimum), have gone through an 8-hour training and certification process for the administration of PRISM, and have practiced delivering the intervention with 2 peers to demonstrate competency. A standardized manual is used to guide PRISM coaches through each session and includes guided discussion prompts to facilitate rapport building and patient participation. Sessions are audio-recorded, and fidelity checks are conducted regularly by a supervisor. There is a standardized fidelity checklist used for scoring purposes and a safety plan to address psychological distress, as needed.

### Participants

This study includes males and females aged ≥12 and <17 years who have a new diagnosis of CMP and are coming in person for a clinic visit, and one of their parents or legal guardians. Exclusion criteria include patients (1) with isolated and localized head pain or abdominal pain, (2) with a diagnosis of complex regional pain syndrome (CRPS), (3) currently receiving CBT, and (4) not capable of understanding the study or providing assent/consent.

### Recruitment and Randomization

**Figure 2:**
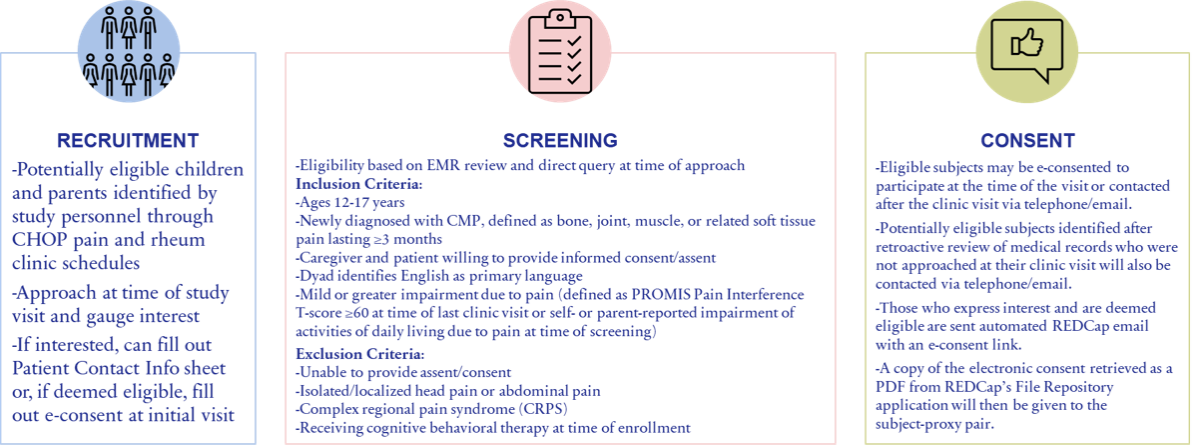
PRISM RCT recruitment, screening, and consent process. CHOP: Children’s Hospital of Philadelphia; CMP: chronic musculoskeletal pain; e-consent: electronic consent; EMR: electronic medical record; PRISM: Promoting Resilience in Stress Management; PROMIS: Patient-Reported Outcomes Measurement Information System; RCT: randomized controlled trial.

Eligible participants are identified by study personnel through screening of the pediatric rheumatology and subspecialty pain clinic schedule via electronic medical records (EMRs). An EMR review is conducted to confirm eligibility, and if further information is needed, an in-person or telephone query is conducted to ensure eligibility. Study personnel approach the eligible patient and a parent or legal guardian at the time of their clinic visit or retroactively via telephone. The study is introduced using the Institutional Review Board (IRB)-approved informed consent form as guidance, and all questions are answered in accordance with IRB regulations. If the patient and their parent express interest but need more time to think about it, they fill out a contact information sheet, are provided with a consent form to take home, and are contacted after the clinic visit via telephone. If eligible and interested, they can provide electronic consent (e-consent) at the time of the clinic visit (Figure 2).

Those who are not approached at their clinic visit can be contacted retroactively via telephone and, if interested and eligible, can be e-consented via REDCap (Research Electronic Data Capture), a secure, web-based software platform designed to support data capture for research studies [24,25]. For anyone who declines participation, their reasoning is recorded in the study’s master log. Study personnel attempt to contact patient-caregiver dyads a maximum of 3 times before discontinuing recruitment efforts. Once baseline surveys are complete, the dyads will be randomly assigned to a study arm using a 1:1 allocation ratio: PRISM+usual care versus usual care.

Stratified randomization will be implemented by the study statistician in REDCap to ensure a balance in sex (male vs female) and clinic (rheumatology clinic vs interdisciplinary pain [amplified musculoskeletal pain] clinic) between the 2 study arms. The final randomization scheme will be prepared by a statistician independent of the study team to prevent bias. Study staff will not get access to randomization algorithms. Tracking of dyads and randomization will be recorded in the study’s master log.

### Medical Record Review and Questionnaires

Each enrolled participant’s medical chart will be reviewed by a study team member for information relevant to the study, including demographics, previous diagnoses, family medical history, previous surgeries, pain-related symptoms, and past treatment. Both members of each dyad will complete questionnaires at 0 months/time of enrollment; 3 months postenrollment or, if in the treatment group, immediately after intervention completion; and 9 months postenrollment. Questionnaires will be completed using an online REDCap survey. Participants will get 45 days to complete each set of questionnaires. Reminder emails or text messages (depending on participant preference) will be sent once a week to ensure completion, and reminder calls will be made a maximum of 3 times. Participants will be compensated for their time and completion of surveys. Caregivers will be paid US $20 for completion of survey 1, US $40 for completion of survey 2, and US $20 for completion of survey 3. Patient participants will be paid US $25 for completion of survey 1, US $50 for completion of survey 2, and US $25 for completion of survey 3. Should subjects request early termination, we will enumerate the next study survey as the final study survey and compensate participants with US $10 for completion of the final surveys. Next, we describe the study measures included in the questionnaires.

The patient measures are as follows:

- Demographics and medical history survey. For the first set of surveys, adolescent participants will be asked whether they have or are currently receiving counseling services and physical therapy, as well as about their exercise routine and whether they perform desensitization exercises.
- The 10-item Connor-Davidson Resilience Scale (CD-RISC-10) [26] measures the overall sense of resilience, or ability to cope with stressors/challenges, and assesses treatment response. Higher scores (0-40) indicate greater resilience. The CD-RISC-10 has good internal consistency and test-retest reliability.
- The Benefit Finding Scale for Children (BFSC) [27] is a 10-item measure of benefit finding designed for children. It is a unidimensional measure and has excellent internal consistency reliability. In a sample of children with cancer, small but significant correlations were found with measures of optimism and self-esteem and a negative correlation with trait anxiety [27].
- The Pain Catastrophizing Scale, Child report (PCS-C) [28] is a validated self-report measure used to assess negative thinking associated with pain. Higher scores reflect higher levels of catastrophic thinking [29].
- The Children’s Hope Scale [30] measures hopeful thought patterns, or the perception that one’s goals can be achieved. Higher scores indicate greater hopeful thought patterns.
- The Children and Adolescent Mindfulness Measure (CAMM) [31-33] is a unidimensional measure of mindfulness. Higher scores indicate higher levels of mindfulness. CAMM has good psychometric properties overall.
- The 8-item Chronic Pain Acceptance Questionnaire Adolescent Short Form (CPAQ-A8) [34] measures pain acceptance using 4 items for each established factor (pain willingness and activity engagement) and is sensitive to treatment. It is correlated with clinically important variables, as its full-length version. Items are scored on a 5-point Likert scale (0=never true to 4=always true). Higher scores indicate more positive perceptions of pain.
- The Pain Self-Efficacy Scale Child (PSES-C) [35] is a 7-item measure assessing child self-efficacy for functioning normally when in pain. Items are scored on a 5-point Likert scale, with lower scores indicating higher self-efficacy. Initial validation has provided support for the PSES-C’s validity and reliability.
- The PROMIS (Patient-Reported Outcomes Measurement Information System) Pediatric Global Health 7 (PGH-7) [36,37] is a brief and reliable 7-item summary assessment of a child’s self-reported health. It represents an individual’s overall assessment of their health, focusing on physical, mental, and social health components. The PGH-7 is well understood by children as young as 8 years of age. The measure has test-retest reliability estimates above 0.80, indicating excellent stability. The measure is unidimensional and factor invariant by the age of the child. Raw scores are converted to T-score (SD) values. The mean score is 50 (SD 10).
- The Functional Disability Inventory (FDI) [38] is a measure of pain-related disability. It is a 15-item measure assessing the degree to which pain interferes with children’s physical functioning over a 2-week period. The FDI has demonstrated adequate psychometric properties in youth with CMP aged 8-18 years [38,39]. The FDI has high internal consistency, moderate-to-high test-retest reliability, and good predictive validity [38-40].
- The PROMIS Pediatric Numeric Rating Scale v1.0 – Pain Intensity [41] provides a numerical value for self-reported pain intensity experienced by the child in the past 7 days.
- The PROMIS Pediatric Bank v2.0 – Pain Interference Short Form 8a [41] measures self-reported consequences of pain.
- The 8-item Patient Health Questionnaire (PHQ-8) [42-46] is a reliable and valid measure of depression severity and a good screening measure in adolescents [43].
- The 7-item Generalized Anxiety Disorder scale (GAD-7) [47,48] has been demonstrated to be a useful screening measure for anxiety severity in adolescents (11-17 years).
- The Kessler-6 Psychological Distress Scale (K6) [49,50] is a 6-item scale that measures the level of psychological distress experienced in the past month. It was developed for the US National Health Interview Survey and is currently being used in Canada, Australia, and the rest of the world as part of the World Health Organization’s (WHO) world mental health initiative. The instrument strongly discriminates between community cases and noncases of *Diagnostic and Statistical Manual of Mental Disorders* (DSM)-IV psychiatric disorders, such as serious emotional distress or serious mental illness (area under the curve [AUC]=0.74-0.88) [51]. The K6 has been extensively cross-validated, including among adolescents and parents. Responses are scored on a 5-point Likert scale (total score ranging from 0 to 24). Previous studies have shown that scores≥7 are consistent with high distress, and scores≥13 meet the criteria for serious or debilitating psychological distress [49].
- The Pain and Symptom Assessment Tool (PSAT) [52] is a modified version of the 2010 American College of Rheumatology (ACR) criteria for fibromyalgia (FM) syndrome, which has demonstrated good specificity and sensitivity for the diagnosis of juvenile FM in adolescents [53]. The PSAT consists of 2 components: the Widespread Pain Index (WPI) and the Symptom Severity (SS) checklist. The WPI is a questionnaire that identifies pain in the past week in 19 body locations (score 0-19). The SS checklist comprises 2 components that identify the severity of cardinal symptoms on a scale from 0 (no problem) to 3 (severe problem) and the presence or absence of associated somatic symptoms. A score is assigned based on the overall number of symptoms (0-3: 0=none to 3=a great deal of symptoms). A final SS score is calculated by adding the cardinal symptoms’ scores to the somatic symptoms’ checklist scores (maximum score=12). According to the adult ACR FM criteria, a score of ≥5 and a WPI of ≥7 or an SS score of ≥9 and a WPI of 3-6 indicates a positive diagnosis for juvenile FM.
- The Acceptability of Intervention Measure (AIM), the Intervention Appropriateness Measure (IAM), and the Feasibility of Intervention Measure (FIM) [54,55] are 4-item measures of implementation outcomes that are often considered leading indicators of implementation success. With readability at the fifth-grade level, these measures can be administered to a wide range of stakeholders to determine the extent to which they believe an intervention or an implementation strategy is acceptable, appropriate, and feasible. The IAM items can be modified to specify a referent organization, situation, or population. Cutoff scores for interpretation are not yet available; however, higher scores indicate greater acceptability, appropriateness, and feasibility. The AIM, IAM, and FIM have demonstrated strong psychometric properties. Specifically, the measures have demonstrated content validity, discriminant content validity, reliability, structural validity, structural invariance, known-groups validity, and responsiveness to change. The predictive validity of the measures is currently being evaluated.
- Satisfaction with PRISM. Session-specific and overall intervention satisfaction will be measured via a 5-point Likert scale (1=very dissatisfied to 5=very satisfied). The intervention will be defined as acceptable if the mean level of satisfaction (including session-specific and overall intervention satisfaction) is ≥3.

The caregiver measures are as follows:

- CD-RISC-10
- K6
- The Pain Catastrophizing Scale – Parent report (PCS-P) [56] measures parental catastrophizing about their adolescent child’s pain. The PCS-P includes 13 items concerning different catastrophic thoughts and feelings parents may have when their child experiences pain. The items are scored using a 5-point Likert-scale (0=not at all to 4=extremely). The PCS-P yields 3 subscale scores for rumination, magnification, and helplessness, as well as a total score ranging from 0 to 52.
- The Adult Hope Scale (AHS) [57] measures Snyder’s cognitive model of hope, which defines hope as a “positive motivational state that is based on an interactivity derived sense of a) agency (goal-directed energy), and b) pathways (planning to meet goals).” The AHS consists of 12 items. Four items measure pathways thinking, four items measure agency thinking, and four items are fillers. Participants respond to each item using an 8-point scale ranging from definitely true to definitely false. When administering the scale, it is called the Future Scale. The agency subscale score is derived by summing items 2, 9, 10, and 12. The pathways subscale score is derived by adding items 1, 4, 6, and 8. The total AHS score is derived by summing the 4 agency and the 4 pathway items.
- The PROMIS Global Health (GH-10) measure version 1.2 [58] is a brief and reliable 10-item summary of an adult’s self-reported physical and mental health. It was developed as part of the US National Institutes of Health’s (NIH) PROMIS initiative using item response theory and rigorously validated in a sample of >20,000 people, primarily from the community. It is freely available and can be used across diseases with T-scores normalized to the US general population. It takes adults no more than 1-2 minutes to complete. Raw scores are converted to T-score (SD) values. The mean score is 50 (SD 10). Higher T-scores reflect more of the trait being measured.

### Ethical Considerations

The study was approved by the CHOP IRB (IRB 22-020664; approval date April 21, 2023; most recent amendment #21 approved on May 8, 2025). The protocol was also reviewed by the National Institute of Arthritis and Musculoskeletal and Skin Diseases (NIAMS) at the NIH. Any further amendments to the study protocol will be communicated to the CHOP IRB and NIAMS at the NIH for formal approval.

Written informed consent will be obtained from all participants, as described in this protocol. All participants will provide informed consent by acknowledging that they have read and understood the consent form before randomization. Information about the study’s significance, purpose, procedures, risks, and benefits will be provided to all eligible patients during the consent process. Identifiable information will be collected from subjects and parent participants only to link the questionnaires and qualitative data from the interviews to the EMR data. Identifiable information linking individual subjects to their PHI (i.e., the master list) will be stored electronically in a Microsoft Excel file located on a secure research SAN drive for the duration of the study and will be maintained in keeping with CHOP policy. Research documents located on the SAN drive will be accessible only to members of the study team. Upon study completion, the database will be maintained in locked mode for a minimum of 3 years, as per regulatory guidelines. Data management procedures are included in our manual of operating procedures, which can be made available upon request.

Since the survey measures ask questions about mood, stressors, psychological symptoms, and feelings, this might put participants at risk for having negative thoughts and emotions. It is not likely that answering these survey measures will create additional distress beyond that which is already present, but research staff will offer referral for clinical services (for child or parent) for any reported distress warranting clinical attention. In addition, if during study participation, the study team discovers abuse or neglect or if threat to harm self or others occurs, they will follow established institutional procedures for mandatory reporting of this information and will provide referrals for appropriate clinical follow-up. We also have a data safety monitoring plan (DSMP) and a data safety monitoring board (DSMB). The DSMB consists of 4 members, who meet once a year to review study progress and safety or confidentiality concerns. In the unlikely event of an adverse outcome associated with this study protocol (eg, disclosure of patient information), it will be documented and discussed with the research team and promptly reported to the local IRB and study sponsor, if appropriate. Any serious adverse events will be reported to the local IRB and the DSMB within 48 hours and to the study sponsor.

### Treatment Group

Patients randomized to the treatment (PRISM+usual care) group will complete the first PRISM session within ~45 days of enrollment. Patients will complete the PRISM sessions remotely using a HIPAA (Health Insurance Portability and Accountability Act)-complaint videoconferencing platform. Each session will be scheduled approximately 1-2 weeks apart but must be completed within 45 days of the previous session. We will invite each patient-parent dyad to participate in an optional 30-minute semistructured interview (see Multimedia Appendix 2 for the interview guide) that will ask about their thoughts regarding PRISM, including background, timing, duration, attendance, content, sessions, and overall satisfaction. Participants will be purposively selected to enrich the cohort for those with early dropout and ensure representation of a variety of ages, races, and ethnicities. These interviews will be conducted by a study staff member other than the PRISM coach, and they will be audio-recorded and transcribed verbatim for qualitative assessment. Participants will be paid US $25 each for their time and completion of semistructured interviews.

### Control Group

**Figure 3:**
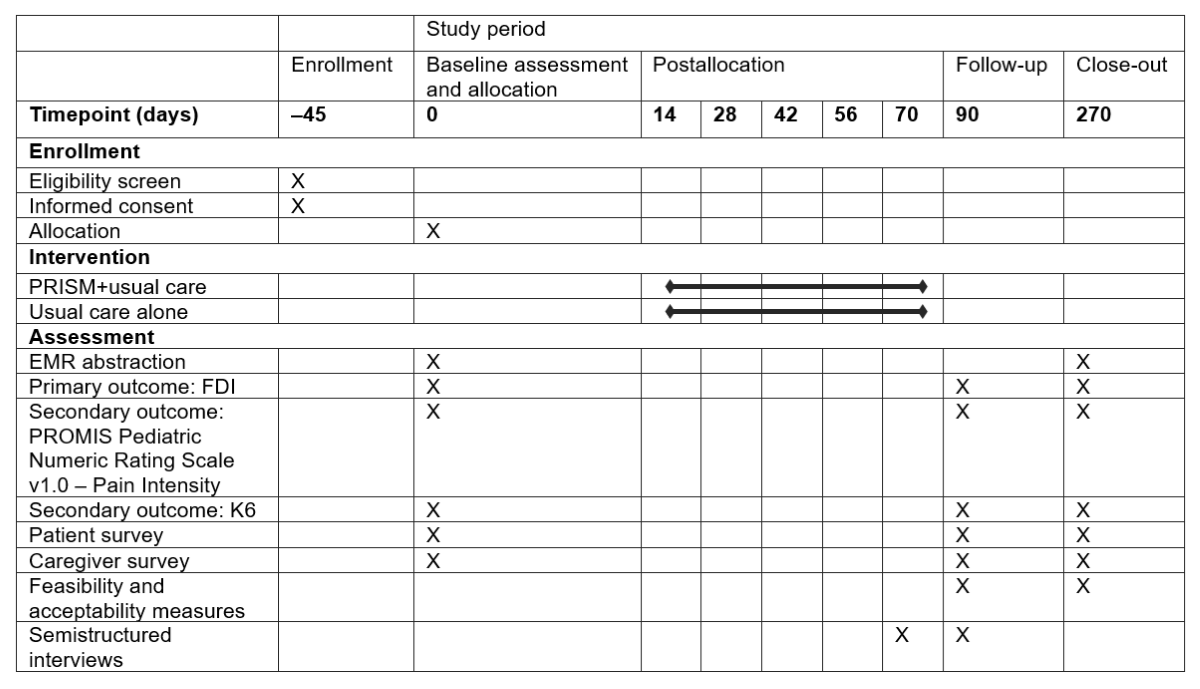
Schedule of study enrollment, interventions, and assessments. EMR: electronic medical record; FDI: Functional Disability Inventory; K6: Kessler-6 Psychological Distress Scale; PRISM: Promoting Resilience in Stress Management; PROMIS: Patient-Reported Outcomes Measurement Information System.

This is the usual care group. This group will be assigned online questionnaires, as described earlier. Control group participants will not receive the PRISM intervention but will receive usual care. Although adolescent participants cannot be actively engaged in CBT at the time of enrollment, they will be allowed to start any type of mental health treatment recommended as part of their treatment plan for their CMP at any point during the study. Additionally recommended as part of usual care will be cardiovascular exercise and desensitization, which may include formal physical and occupational therapy. See Figure 3 for a comprehensive description of study visit activities and the timeline for both study arms.

### Statistical Analysis

The primary objective of this study is to determine whether PRISM, along with usual care, reduces functional disability more than usual care alone. The primary outcome or measure of efficacy is the FDI score at 3 months postrandomization (control group) or postintervention. The secondary efficacy outcomes are patient psychological distress (as measured with the K6), as well as the patient-reported pain intensity (as measured with the PROMIS Pediatric Numeric Rating Scale v1.0 – Pain Intensity). The secondary objectives are to determine a reduction in pain intensity and psychological distress with PRISM at intervention completion; determine reductions in functional disability, pain intensity, and psychological distress with PRISM at 6 months postintervention; evaluate potential patient- and caregiver-level moderators of PRISM; examine implementation outcomes (feasibility, acceptability, appropriateness); and identify facilitators of and barriers to engagement in PRISM.

The study team plans to publish the study findings in a peer-reviewed pediatrics, psychology, or rheumatology journal to disseminate the findings. No individually identifiable protected health information will be published. Summary results will be available on ClinicalTrials.gov.

#### Quantitative Data

Quantitative measures will be used to assess the primary and secondary efficacy outcomes, moderators of PRISM, and implementation outcomes. Demographics and baseline clinical characteristics will be summarized by standard descriptive summaries (eg, means and SDs for continuous variables, such as age, and percentages for categorical variables, such as gender). The primary analysis will use an intention-to-treat (ITT) approach to mitigate confounding from nonrandom participant attrition. We anticipate a total of 100 evaluable subjects. To ensure valid randomization, we will assess demographic and clinical features, along with baseline measures of outcomes, such as physical function, pain intensity, and psychological distress, and compare the intervention to the control arm. The primary outcome will be functional disability (FDI score) assessed at the end of the intervention (month 3). The key predictor is PRISM. Secondary outcomes will include the FDI score at the conclusion of the observation period (month 9, 6 months postintervention) and psychological distress (K6 total) and pain intensity (PROMIS Pediatric Numeric Rating Scale v1.0 – Pain Intensity) at both 3 and 9 months.

Assuming balanced arms postrandomization, we will evaluate the differences in clinical outcomes between the 2 arms at months 3 and 9 using 2-sample Student *t* tests. Longitudinal changes from month 3 to month 9 will be assessed using MANOVA (multivariate analysis of variance) and linear mixed effects models to account for within-subject correlation due to repeated measures. The linear mixed effects model can additionally adjust for a few known prognostic covariates, such as fulfillment of criteria for juvenile FM. Similar analyses will examine outcomes of psychological distress and pain intensity separately.

Linear regression will be conducted to assess the association between the change in FDI scores and changes in the intervention targets, adjusted for baseline covariates of interest. These analyses will be repeated to examine correlations between changes in secondary outcomes (pain intensity and psychological distress) and hypothesized intervention targets at month 3, and we will conduct multivariable linear regression models, adjusting for covariates of interest.

Furthermore, we will explore adolescent- and caregiver-level moderators of intervention effects, guided by an intersectionality framework [59]. For instance, we will investigate whether age moderates the intervention’s impact on functional disability by including the age × intervention interaction in the linear mixed effects regression model. If the interaction is significant, we will interpret it by plotting simple regression lines for younger (12-14 years) and older (15-16 years) adolescents. If PRISM proves more beneficial for younger participants, future studies may focus on this age group to maximize intervention effects. Additionally, we will evaluate whether biological sex moderates the intervention’s impact, ensuring adequate inclusion of male participants, given the predominance of females in the population with CMP [60].

Analyses will include calculation of eligibility, recruitment and attrition rates, and power considerations for the future full-scale RCT and evaluation of treatment fidelity. We will report the number of eligible subjects approached, the proportion of those subjects enrolled in the study, and the mean percentage of required sessions (PRISM 1-4 sessions + “coming together” session) attended among enrolled subjects. The study will be defined as feasible (binary) if the recruitment rate is ≥60%, the completion rate of all sessions among enrolled subjects is ≥70%, and the mean FIM score is ≥4. The intervention will be acceptable and appropriate if the mean AIM and IAM scores are ≥4 each. Intervention satisfaction (including session-specific and overall intervention satisfaction) will be reported as mean scores from the 5-point Likert scale (1=very dissatisfied to 5=very satisfied).

With a sample of 130 patient participants and an assumed ~20% attrition rate, we have more than 80% statistical power to detect a difference down to 5 points in the FDI between the intervention and control arms, given a prespecified mean FDI score of 26.9 (SD 11.8) and a significance level of .05 for the Student *t*-test. Table 1 lists the detectable differences (DDs) for the outcomes of interest, as well as the minimal clinically important difference (MCID) for each measure. The effect sizes of CBT on the FDI in similar patient populations have been strong to moderate (0.8-0.9), with between-group differences in the FDI score of 5.39 (95% CI 1.57-9.22). Therefore, we will be adequately powered for the primary outcome [61,62].

**Table 1:** DDs^a^ in mean instrument scores between groups, given 80% power and a 5% type I error rate (N=100^b^).

Outcome (instrument)	Sample mean (SD)^c^	MCID^d^	DD
Physical function (FDI^e^)	26.9 (11.8)	8.0	5.0
Pain (PROMIS^f^ Pediatric Numeric Rating Scale v1.0 – Pain Intensity)	6.2 (2.3)	2.0	0.9
Psychological distress (K6^g^)	9.1 (6.0)	3.0^h^	2.4

^a^DD: detectable difference.

^b^Anticipated dropout rate of ~20% (total=130), resulting in 100 evaluable subjects.

^c^Sample means from preliminary data.

^d^MCID: minimal clinically important difference.

^e^FDI: Functional Disability Inventory.

^f^PROMIS: Patient-Reported Outcomes Measurement Information System.

^g^K6: Kessler-6 Psychological Distress Scale.

^h^Half the SD of mean baseline scores.

#### Qualitative Data

Semistructured interview findings will be used to understand barriers and facilitators to support future implementation of PRISM. Three team members will conduct the interviews after completing training on interview facilitation best practices. Research team members have a diverse experience spanning the domains of chronic pain, rheumatology, patient-centered care, and qualitative research methods and have varied levels of preknowledge of CMP. The principal investigator’s interpretation of interview findings will be informed by their expertise in treating adolescents with CMP, while several other team members have prior knowledge of CMP through their research work. To maximize reflexivity, the research team members will discuss their assumptions about the research topics and how these might influence their interpretation of findings both before and during data analysis.

Interviews with patients and caregivers will be transcribed verbatim. Two members of the study team will code transcripts independently and meet to identify emergent themes arising from a line-by-line review of narrative content, as well as a priori codes derived from the Consolidated Framework for Implementation Research (CFIR) and the Assessment, Decision, Adaptation, Production, Topical experts, Integration, Training and Testing (ADAPT-ITT) framework [63]. Next, 20% of interviews will be double-coded to insure intercoder reliability of the developed coding schema and enhance credibility of findings. Discrepancies will be resolved through consensus. Quantitative findings on efficacy and implementation outcomes will be synthesized with qualitative findings on facilitators of and barriers to engagement to support wide-based dissemination of PRISM in a multicenter RCT.

## Results

The trial is currently open. Initial IRB approval was obtained on April 21, 2023, and protocol version 4 was amended on January 14, 2025. Recruitment began on May 8, 2023, and we anticipate recruitment completion on August 1, 2025.

## Discussion

### Summary

This pilot RCT protocol is designed to assess whether the addition of PRISM to usual care is associated with greater improvements in functional disability compared to usual care alone. This intervention has the potential to enhance pain management and overall well-being for youth with CMP, who have been shown to have lower resilience than unaffected adolescents. The results of this study will heighten our understanding of the role of resilience over time and shed light on strategies that promote psychological resilience for patients with CMP and their caregivers. These insights will lay the groundwork for multicenter studies on the efficacy of PRISM in CMP and inform future treatment strategies for this population.

### Limitations

In terms of limitations of this protocol, it may be lacking in generalizability, as the population with CMP at our institution has been limited in racial and ethnic diversity. However, we do expect that our study sample will reflect the demographic makeup of patients with CMP within our clinic population. It is unclear based on the current literature whether our patient population aligns demographically with the population with CMP nationally. Our protocol is also limited to English-speaking participants. Offering PRISM in other languages would further increase the generalizability of future studies. Additionally, although pilot trial participants have approved of our flexibility in scheduling PRISM sessions outside of normal business hours, this may not be feasible in real-world clinical settings. Similarly, the 1-on-1 format of the intervention may be too time and resource intensive for routine clinical practice. This emphasizes the importance of future research to address these limitations and guide future implementation efforts.

### Conclusion

Findings from this work will further enhance our understanding of psychosocial factors that play an important role in adolescent CMP, with the goal of reducing the disease burden and improving long-term outcomes. A future multicenter RCT of PRISM for adolescents with CMP will provide further evidence of the efficacy of this program and inform clinical implementation. For some adolescents, the addition of PRISM to routine clinical care could offer sufficient pain-coping skills to reduce their need for individual counseling. For others, these learned skills may serve as a critical interim until formal CBT becomes available. Although PRISM would not fully solve issues such as clinic wait times, the integration of PRISM into clinical care could help prioritize the limited appointments in pediatric chronic pain clinics for those with the greatest need for interdisciplinary care.

## Appendix

Multimedia Appendix 1 SPIRIT (Standard Protocol Items: Recommendations for Interventional Trials) checklist.

Multimedia Appendix 2 Semistructured interview guide.

Multimedia Appendix 3 Peer-review report from AMS - Arthritis and Musculoskeletal and Skin Diseases Special Grants Study Section, National Institute of Arthritis and Musculoskeletal and Skin Diseases (National Institutes of Health, USA).

## Abbreviations

ACR: American College of Rheumatology
AHS: Adult Hope Scale
AIM: Acceptability of Intervention Measure
CAMM: Children and Adolescent Mindfulness Measure
CBT: cognitive behavioral therapy
CD-RISC-10: 10-item Connor-Davidson Resilience Scale
CHOP: Children’s Hospital of Philadelphia
CMP: chronic musculoskeletal pain
DSMB: data safety monitoring board
e-consent: electronic consent
EMR: electronic medical record
FDI: Functional Disability Inventory
FIM: Feasibility of Intervention Measure
FM: fibromyalgia
IAM: Intervention Appropriateness Measure
IRB: Institutional Review Board
K6: Kessler-6 Psychological Distress Scale
MCID: minimal clinically important difference
NIAMS: National Institute of Arthritis and Musculoskeletal and Skin Diseases
NIH: National Institutes of Health
PCS-C: Pain Catastrophizing Scale, Child report
PCS-P: Pain Catastrophizing Scale, Parent report
PGH-7: Pediatric Global Health 7
PRISM: Promoting Resilience in Stress Management
PROM: patient-reported outcome measure
PROMIS: Patient-Reported Outcomes Measurement Information System
PSAT: Pain and Symptom Assessment Tool
PSES-C: Pain Self-Efficacy Scale Child
RCT: randomized controlled trial
REDCap: Research Electronic Data Capture
SS: Symptom Severity
WPI: Widespread Pain Index

## Data Availability

Data sharing is not applicable to this paper as no datasets will be generated or analyzed during the study.
