# Supplementary material for: Resilience Coaching for Adolescent Chronic Musculoskeletal Pain: Protocol for a Pilot Randomized Controlled Trial of Promoting Resilience in Stress Management (PRISM)
Source: JMIR Res Protoc. 2025 Jul 22;14:e73385. doi: 10.2196/73385 (PMC12326160; doi:10.2196/73385)
Supplement: Multimedia Appendix 2 [file resprot_v14i1e73385_app2.docx]

**A. Semi-Structured Interview Guide for Patients**

Qualitative interview introduction: First, I just want to say thank you for participating in this study. This semi-structured interview will take about 20-30 minutes. I am going to ask questions in order to get feedback regarding the resilience coaching program, PRISM. For example, to see what you did and didn’t like about it so we can make it better for future participants.

Verbal assent: You and your parent (or legal guardian) already formally agreed to participate in this study (assent and consent, respectively) but we would like to ask you again, if you agree to participate. This conversation will be recorded and you can request to stop or pause the recording at any point. Do I have your permission? Please tell me your name and date of birth to confirm your identity.

1. Background information [CFIR Domain(s): Characteristics of the Intervention / Characteristics of the Individual / Inner Setting / Outer Setting / Process; ADAPT-ITT Domain(s): Assessment / Decision / Adaptation]

a. Please tell me about your experience with the resilience coaching program. [Looking for general information and mostly about experiences and perspectives of PRISM]

b. Now please describe how you think PRISM could be helpful for people with chronic musculoskeletal pain.

i. What did you find helpful about PRISM?

ii. What do you think was missing from PRISM that young people dealing with chronic pain might need? What could have worked better?

c. Why did you decide to participate in PRISM? How do you feel about it now that you have completed the program? What would you say to a teen dealing with chronic pain who might be on the fence about participating in PRISM?

2. Timing [CFIR Domain(s): Characteristics of the Intervention / Characteristics of the Individual; ADAPT-ITT Domain(s): Assessment / Decision / Adaptation]

a. How did the timing (how long and when the sessions were held) of PRISM work for you?

b. How do you think we could adjust the timing to work better for you or other teens dealing with chronic pain?

3. Duration [CFIR Domain(s): Characteristics of the Intervention; ADAPT-ITT Domain(s): Assessment / Decision / Adaptation]

a. What do you think about the number of sessions? Does it need fewer or more? What would you spend less time on/cut out (if less)? // What would you spend more time on/what would you add (if more sessions)?

b. What do you think about the length of the sessions? Do they need to be longer or shorter?

c. What do you think about how frequently they were scheduled? What is the right amount of time for you to practice and work on the skills but still feel like you are connected to the program?

4. Attendance [CFIR Domain(s): Characteristics of the Intervention / Characteristics of the Individual / Inner Setting / Outer Setting / Process; ADAPT-ITT Domain(s): Assessment / Decision / Adaptation]

a. What got in the way of attending PRISM sessions?

b. What would have helped you or others from missing sessions?

5. Virtual format

a. How did the Microsoft Teams meetings work for you? Did you use a phone or computer for sessions? How was the process of logging in to meetings? What did you think about the call quality? What did you think about your coach sharing their screen with you to share cheat sheets?

b. What programs do you prefer to use for video calls (e.g., Zoom, Skype, Facetime, etc.)?

c. How could we make the video call aspect of PRISM work better?

6. Content [CFIR Domain(s): Characteristics of the Intervention / Characteristics of the Individual; ADAPT-ITT Domain(s): Assessment / Decision / Adaptation]

a. What information or topics did you find the most helpful in PRISM? How were they helpful?

b. What topics weren’t as helpful?

c. What topics could we add that would be helpful? (Probe: What help do people need in this area?)

d. What skills did you learn from PRISM? (Probe: How do you think you will use these skills in the future?)

e. Did you have any previous knowledge about any of these skills?

i. Which skills have you done before?

ii. Did you think it was a good refresher/was there anything new about it that you learned/built upon?

7. Session-Specific feedback [CFIR Domain(s): Characteristics of the Intervention / Characteristics of the Individual; ADAPT-ITT Domain(s): Assessment / Decision / Adaptation]

a. Now I wanted to talk to you a bit about the session we had with your parent (or legal guardian).

i. How did you feel about the session with your parent (or legal guardian)? (probe: What did you like? What did you not like?)

ii. How do you feel about your parent (or legal guardian) knowing about what you were learning in PRISM? (probe: How could your parents’ experience with PRISM be helpful to you dealing with your chronic pain?)

ii. How did you feel about the number of sessions with your parent (or legal guardian) compared to the individual sessions?

iii. What would you change about the session with your parent (or legal guardian) to help them feel more useful?

8. Overall satisfaction [CFIR Domain(s): Characteristics of the Intervention / Characteristics of the Individual; ADAPT-ITT Domain(s): Assessment / Decision / Adaptation]

a. On a scale of 1-5 with 1 indicating very unlikely and 5 indicating very likely, how likely would you be to recommend PRISM to other patients? (Probe: Why did you rate it that number? What kept it from being a 5 for you?)

b. Would you recommend PRISM to another patient? Why or why not?

c. We want to make PRISM the best we can for young people with chronic pain. If you could create your own version of PRISM or something like it, what would it look like or how would it be different from our version?

d. Previous participants have shared with us their interest in having one or more group sessions or other opportunities for interaction with other young people with chronic pain. What are your thoughts on this?

1. How would you design group sessions to make them most useful?
   1. How many; how often?
   2. In person; virtual?
   3. Structured; free form?
2. What would you get out a group session that you wouldn’t from an individual session?
   1. What would be the best way to deliver those skills/goals for you?
3. What kinds of interactions with peers or resilience coaching would you like to have after the completion of PRISM?

9. Last thoughts [CFIR Domain(s): Characteristics of the Intervention / Characteristics of the Individual / Inner Setting / Outer Setting / Process; ADAPT-ITT Domain(s): Assessment / Decision / Adaptation]

a. Tell me anything else that you would like to share with me regarding PRISM.

b. Tell me any last thoughts on what was helpful or what was missing from PRISM.

c. How did participating in PRISM makes you think differently about working with a psychologist/mental health professional. (Probe: stigma; openness; motivation for seeking mental health services)

i. Now that you’ve participated in PRISM, how would you think about continuing to work on resilience with a psychologist or mental health professional?

ii. How does knowing that PRISM is based in CBT or cognitive behavioral therapy change your interest or decision to participate?

10. Early termination [CFIR Domain(s): Characteristics of the Intervention / Characteristics of the Individual / Inner Setting / Outer Setting / Process; ADAPT-ITT Domain(s): Assessment / Decision / Adaptation]

a. As we try to make this program better, it would be helpful for us to know why you decided to [withdraw consent to participate in this study] *or* [end this study early]? Could you please tell me why you decided this?

Closing: Thank you so much for participating and giving me your honest feedback on PRISM. This is the end of the interview so I will stop recording now.

**B. Semi-Structured Interview Guide for Caregivers**

Qualitative interview introduction: First, I just want to say thank you for participating in this study. This semi-structured interview will take about 20-30 minutes. I know you did not yourself take part in the resilience coaching, but as a caregiver, you were still greatly involved. I am going to ask questions in order to get your feedback regarding the resilience coaching program, PRISM. For example, to see what you did and didn’t like about it so we can make it better for future participants.

Verbal assent:

You already formally agreed to participate in this study but we would like to ask you again, if you agree to participate. This conversation will be recorded and you can request to stop or pause the recording at any point. Do I have your permission? Please tell me your name and date of birth to confirm your identity.

1. Background information [CFIR Domain(s): Characteristics of the Intervention / Characteristics of the Individual / Inner Setting / Outer Setting / Process; ADAPT-ITT Domain(s): Assessment / Decision / Adaptation]

a. Please briefly tell me about your experience with the resilience coaching program. [This would include general information and mostly about experiences and perspectives of the resilience coaching program]

b. Why did your child decide to participate in PRISM? How do you feel about it now that he/she completed the program?

2. Timing [CFIR Domain(s): Characteristics of the Intervention / Characteristics of the Individual; ADAPT-ITT Domain(s): Assessment / Decision / Adaptation]

a. How did the timing (how long and when the sessions were held) of PRISM work for

you and your child?

b. How do you think we could adjust the timing to work better for your child or other teens with chronic pain?

3. Duration [CFIR Domain(s): Characteristics of the Intervention; ADAPT-ITT Domain(s): Assessment / Decision / Adaptation]

a. What do you think about the number of sessions? Does it need fewer or more?

b. What do you think about the length of the sessions? Do they need to be longer or shorter?

c. What do you think about how frequently they were scheduled? What is the right amount of time for you to practice and work on the skills?

4. Attendance [CFIR Domain(s): Characteristics of the Intervention / Characteristics of the Individual / Inner Setting / Outer Setting / Process; ADAPT-ITT Domain(s): Assessment / Decision / Adaptation]

a. What got in the way of your child attending PRISM sessions?

b. What would have helped your child with attending the sessions?

5. Virtual format

a. How did the Microsoft Teams meetings work for you and your child? Did your child use a phone or computer for sessions? How was the process of logging in to meetings? What did you think about the call quality? What did you think about the coach sharing their screen with you to share cheat sheets?

b. What programs do you prefer to use for video calls (e.g., Zoom, Skype, Facetime, etc.)?

c. How could we make the video call aspect of PRISM work better?

6. Content [CFIR Domain(s): Characteristics of the Intervention / Characteristics of the Individual; ADAPT-ITT Domain(s): Assessment / Decision / Adaptation]

a. What did you think about the topics and information included in the intervention? How were they helpful?

b. Were there any topics that you didn’t like? Why?

c. Is there anything that you would add to PRISM?

7. Session-Specific feedback [CFIR Domain(s): Characteristics of the Intervention / Characteristics of the Individual; ADAPT-ITT Domain(s): Assessment / Decision / Adaptation]

a. Now I wanted to talk to you a bit about the session we had with you and your child.

i. How did you feel about the session with your child? (probe: What did you like? What did you not like?)

ii. How do you feel about your child sharing with you what they are learning in PRISM?(probe: How else could you envision your involvement in PRISM helping your child deal with their pain?))

ii. How did you feel about the number of sessions with your child compared to the individual sessions?

iii. What would you change about the session with your child to help them feel more useful?

8. Overall satisfaction [CFIR Domain(s): Characteristics of the Intervention / Characteristics of the Individual; ADAPT-ITT Domain(s): Assessment / Decision / Adaptation]

a. On a scale of 1-5 with 1 indicating very unlikely and 5 indicating very likely, how likely would you be to recommend PRISM to other patients/parents? (Probe: Why did you rate it that number? What kept it from being a 5 for you?)

b. Would you recommend PRISM to another patient/parents? Why or why not?

c. We want to make PRISM the best we can for young people with chronic pain. If you could create your own version of PRISM or something like it, what would it look like or how would it be different from our version?

d. Previous participants have shared with us their interest in having one or more group sessions or other opportunities for interaction with other young people with chronic pain. What are your thoughts on this?

1. How would you design group sessions to make them most useful for teens?
   1. How many; how often?
   2. In person; virtual?
   3. Structured; free form?
2. What would your child get out a group session that they might not from an individual session?
   1. What would be the best way to deliver those skills/goals for your child?
3. What kinds of interactions with peers or resilience coaching resources would you like your child to have after the completion of PRISM?

9. Last thoughts [CFIR Domain(s): Characteristics of the Intervention / Characteristics of the Individual / Inner Setting / Outer Setting / Process; ADAPT-ITT Domain(s): Assessment / Decision / Adaptation]

a. Tell me anything else that you would like to share with me regarding PRISM.

b. Tell me any last thoughts on what was helpful or what was missing from PRISM.

c. Would you be interested in receiving resilience coaching yourself?

10. Early termination [CFIR Domain(s): Characteristics of the Intervention / Characteristics of the Individual / Inner Setting / Outer Setting / Process; ADAPT-ITT Domain(s): Assessment / Decision / Adaptation]

a. As we try to make this program better, it would be helpful for us to know why your child decided to [withdraw consent to participate in this study] *or* [end this study early]? Could you please tell me?

Closing: Thank you so much for participating and for your feedback on PRISM. This is the end of the interview so I will stop recording now.
