# Supplementary material for: Resilience Coaching for Adolescent Chronic Musculoskeletal Pain: Protocol for a Pilot Randomized Controlled Trial of Promoting Resilience in Stress Management (PRISM)
Source: JMIR Res Protoc. 2025 Jul 22;14:e73385. doi: 10.2196/73385 (PMC12326160; doi:10.2196/73385)
Supplement: Multimedia Appendix 3 [file resprot_v14i1e73385_app3.pdf]

1K23AR081409-01 GMUCA, SABRINA

RESUME AND SUMMARY OF DISCUSSION: This is a new application for a K23 Mentored Patient-Oriented Research Career Development Award from Sabrina Gmuca, MD at the Children's Hospital of Philadelphia. The candidate proposes to conduct a pilot randomized controlled trial (RCT) of resilience coaching for adolescents with chronic musculoskeletal pain. The mentoring team is outstanding. The mentor is Dr. Pamela Weiss who is an expert in the field and has strong research support and an excellent mentoring track record. The committee agrees that the candidate is productive and has an outstanding training background in pediatric rheumatology and clinical research with strong letters of support. The career development plan is comprehensively structured and includes an evaluation plan and training in resilience, analytic skills in clinical trials and pediatric chronic pain management. The research environment is exceptional and institutional commitment is strong. The research plan to assess the pain related clinical outcomes will have the potential for high impact on pain management in pediatric and adolescent care. It includes an innovative mediation analysis approach. Only a few issues are noted in the study design. There are some minor concerns regarding the qualitative data analysis, mixed methods, and assumption of retention number in the study. The application could include a discussion of the potential recruitment challenges in an RCT. Overall, the committee concludes that the candidate has high potential to develop into a productive independent scientist.

DESCRIPTION (provided by applicant): Chronic musculoskeletal pain (CMP) affects as many as 4 in 10 children, mostly adolescents, and accounts for the majority of new referrals to pediatric rheumatology. Long-term physical and psychosocial outcomes for adolescents with CMP are poor with increased healthcare utilization and psychological co-morbidities. Additionally, current treatment regimens for CMP have limited accessibility. Resilience is a dynamic process of positive adaptation or development in the context of significant adversity, such as living with excessive chronic pain. Promoting Resilience in Stress Management (PRISM) is a successful resilience-training intervention for adolescents and young adults with serious illness. Preliminary data for this proposal demonstrate a moderate correlation between self-perceived resilience and symptom severity among adolescents as well as high feasibility and acceptability of PRISM delivery among youth with CMP. Qualitative feedback was universally positive with the consistent recommendation to add a patient group session to the intervention. With an added patient group session to the intervention, we now aim to ascertain the impact of the adapted intervention (Promoting Resilience in Stress Management (PRISM) for adolescent Chronic musculoskeletal pain [PRISM-C]) on pain-related clinical outcomes in a randomized controlled trial. The overall objectives of the proposed project are to (1) determine the efficacy of PRISM-C in a population of youth with CMP, (2) explore moderators of the intervention to identify youth most likely to benefit from resilience coaching, and (3) assess implementation outcomes and identify barriers and facilitators to engagement in PRISM-C. Findings from this work will further our understanding of psychosocial factors important in adolescent CMP in order to reduce disease burden and improve long-term outcomes. Study results will serve as preliminary data for an R01 application to perform a multicenter randomized controlled trial of PRISM-C for adolescents with CMP. The proposed Career Development Award addresses how to improve access to and efficacy of interdisciplinary treatments for CMP. The exceptional resources and institutional support at Children's Hospital of Philadelphia and the University of Pennsylvania, outstanding multidisciplinary mentorship team, and proposed career development activities will allow the candidate to achieve her long-term goal of becoming an independent investigator and nationally recognized pediatric rheumatologist with expertise in pediatric chronic pain, resilience, and behavioral health interventions with the goal of improving the long-term physical and psychosocial outcomes for adolescents with CMP.

PUBLIC HEALTH RELEVANCE: There is a critical need to improve the long-term physical and psychosocial outcomes of adolescents with chronic musculoskeletal pain (CMP) in order to reduce healthcare utilization and mitigate the psychosocial sequelae of ongoing chronic pain in this vulnerable

patient population. The goal of the proposed research is to examine the impact of a resilience coaching program adapted for adolescent chronic musculoskeletal pain in a pilot randomized controlled trial (RCT). Achievement of the study aims has the potential to be a significant contribution to building and fostering resilience among adolescents with chronic musculoskeletal pain, as the proposed research will inform a future multicenter RCT of the resilience coaching program and thereby augment current interdisciplinary management of adolescent CMP.

CRITIQUE 1

Candidate: 1
Career Development Plan/Career Goals: 1
Research Plan: 3
Mentor(s), Co-Mentor(s), Consultant(s), Collaborator(s): 1
Environment Commitment to the Candidate: 1

Overall Impact: This is a new mentored patient-oriented research career development award application by PI Dr, Sabrina Andrea Gmuca from the Children's Hospital of Philadelphia. The candidate proposes a pilot randomized controlled trial of resilience coaching for adolescents with chronic musculoskeletal pain. Three well connected specific aims will be achieved in this study: 1). Evaluate the impact of PRISM-C on pain related clinical outcomes in a randomized controlled trial (n = 120) at 3 and 9 months; 2). Explore potential patient and caregiver level of moderators of PRISM-C, and 3). Examine implementation outcomes (feasibility, acceptability, appropriateness) and identify facilitators and barriers via survey and a semi-structured interview. The research is significant, since adolescent chronic pain affect a large percent of adolescents (10 to 30%). Access to treatment is challenging and service is limited with cognitive behavioral therapy, such as resilience coaching for a dynamic process of positive adaptation of continued development in the context of adversity. The candidate is outstanding and has been working on qualitative research which led to this research proposal. The candidate has developed a comprehensive career development plan with details on the training content, evaluation and mentor expertise. The mentor team is strong and comprehensive. The institutional environment and support are also outstanding. Scientist rigors are very good, and sex as a biological variable is considered. There are minor weaknesses with the research approach, but these do not significantly affect the overall high quality of the application.

1. Candidate:

Strengths

- The candidate is assistant professor in the department of pediatrics and attending physician in the division of pediatric rheumatology. She received MD (2011) from the State University of New York Downstate College of Medicine, Brooklyn, NY, did residency (2014) in pediatrics, fellowship (2017) in pediatric rheumatology. She also received M.S.C.E. in Clinical Epidemiology (2018) from Perelman School of medicine at the University of Pennsylvania. The candidate's training background is strong and appropriate for the proposed research and training.
- The candidate has a faculty appointment with the center for Amplified musculoskeletal pain syndrome and center for pediatric clinical effectiveness and Policy Lab at CHOP. She is well posited to take on the proposed project.

- The candidate has been a leadership role (vice chair) of the early investigator committee for childhood arthritis & rheumatology research alliance; was a recipient of Rheumatology Research Foundation K-Bridge Award and is PI for RRF investigator award exploring resilience in adolescent chronic musculoskeletal pain (2020-2023). The candidate has 14 publications, and she is the lead author in 12 of them.
- Overall, this candidate has strong training background and research experience in the areas of research proposed in this application. The candidate is very productive and showed a strong commitment for a patient-centered research career.
- Reference letters are overwhelmingly positive, indicating strong commitments to support the proposed training and research; and suggesting great potential of the candidate for a successful research career.

Weaknesses

- None noted

2. Career Development Plan/Career Goals & Objectives:
Strengths

- There long-term and short and medium-term career goals were well connected in the career development plan. The explicit long-term goal of the candidate's career development is to become a renowned clinician scientist with expertise in the treatment and management of chronic musculoskeletal pain (CMP). Short and medium-term career goals are 1. Develop analytic skills in clinical trials research for larger and complex studies; 2. Enhance knowledge of pediatric chronic pain management and role of resilience; 3. Solidify expertise in mixed-methods, intervention adaptation and refinement, and implementation science that led to continuing professional development and successful grant application.
- The candidate will work with the mentor and co-mentors close during the award, and the mentoring team will meet quarterly, and advisors will join them twice per year. This plan ensures communications among the entire mentoring team. Written evaluations will be obtained from each mentor, co-mentor and advisor twice a year and the mentor will have communication with each co-mentor every other week for feedback. This evaluation plan is excellent to monitor and guide the candidate's career development.
- Course work, workshops, seminars and guided readings and teaching were planned to support the candidate's career development. These activities are well selected with strong rationales to allow the candidate to move from current mentored research to independent research. The candidate will spend at least 75% effort on research.

Weaknesses

- None noted

3. Research Plan:
Strengths

- The candidate's solid qualitative work on PRISM-C led to the development of the K23 project, which is a natural progression of the candidate's research career.

- The research is innovative with mediation analysis to understand mediators of success with the resilience coaching, and to develop strategies for improving individual outcomes with the intervention (personalization).
- The study will use patient-caregiver dyads in a randomized trial design which is very appropriate to the study questions.
- Both the short-term effect (3 month) and the sustainability of the effect (9 month) will be studied to provide better understanding on the impact of the intervention.
- Attrition is considered in sample size estimation.
- Fidelity of coaches will be assessed from a random select session of the five sessions of intervention.
- Multiple questionnaires (demographic, resilience, benefit-finding, hope, mindfulness, self-efficacy, pain-catastrophizing, pain acceptance, functional disability, psychological distress, pain intensity, HRQoL, depression, anxiety, juvenile fibromyalgia criteria, feasibility, acceptability, appropriateness, satisfaction) will be used to collect data on outcomes and potential mediators. This approach provides comprehensive data to inform future research.

Weaknesses

- The qualitative data analysis approach is not very clear. How to use the child and care-giver data, integrative or separate?
- The mixed methods approach needs more consideration on the framework, theory and integration.
- Many questionnaires will be used. What is the total time needed for completing the questionnaires? The participants will receive three set of questionnaires. What is the time interval? Attention should be given to the sequence of the questionnaires.

4. Mentor(s), Co-Mentor(s), Consultant(s), Collaborator(s):

Strengths

- The mentor team is strong. Primary mentor Dr. Pamela Weiss has similar training background as the candidate (pediatrics rheumatology and clinical epidemiology) and is experienced with early diagnosis, accurate phenotyping and targeted treatment of children with spondylosis arthritis. Dr. Weiss has been a long-term mentor to the candidate (since 2014) and instrumental for the candidate's career development at CHP. Co-mentor Jami Young who also comes from CHP is a clinical psychologist and a leader in adolescent psychiatry and behavior sciences at CHP. The other co-mentors also have unique expertise needed for the candidate's training. Dr. Tonya Palermo is a pediatric psychologist. Dr. Abby Rosenberg is co-developer of PRISM and has published a paper with the candidate on how to develop, test, adapt and disseminate chronic musculoskeletal pain (PRISM-C). Both Drs. Palermo and Rosenberg work at the Seattle Children's Hospital.
- In addition to mentors, two advisors are included to provide technical assistance. Dr. Peter Cronholm the patient centered outcomes advisor from University of Pennsylvania will be help the candidate for qualitative research, mixed methods and implementation science; and Dr. Rui Xiao is bio-statistical advisor.

- The primary mentor is well funded and has an excellent track of record training young investigators.
- The supervision and mentoring plan is developed with details on content and procedure.
- Plans for progression to independence and monitoring productivity are in cohesive with the training and evaluation plan from the candidate.
- There are strong commitment from the mentor, co-mentors, and advisors.

Weaknesses

- None noted

5. **Environment:**

Strengths

- There are strong institutional research environment and support.
- Dr. Joseph W. St, Geme, II, MD, is the candidate's Department chair and supports 75% protected research effort of the candidate during the K23 award.

Weaknesses

- None noted

Study Timeline:

Strengths

- Study timeline has taken preparation and data analysis into consideration.
- Study timeline is supported by previous recruitment experience

Weaknesses

- None noted

Protections for Human Subjects:

Acceptable Risks and Adequate Protections

- Minimal risk

Data and Safety Monitoring Plan (Applicable for Clinical Trials Only):

Acceptable

- The data safety monitoring board will meet annually.

Inclusion Plans:

- Sex/Gender: Distribution justified scientifically
- Race/Ethnicity: Distribution justified scientifically
- For NIH-Defined Phase III trials, Plans for valid design and analysis:
- Inclusion/Exclusion Based on Age: Distribution justified scientifically
- Age 12-17

Vertebrate Animals:

Not Applicable (No Vertebrate Animals)

Biohazards:

Not Applicable (No Biohazards)

Training in the Responsible Conduct of Research:
Acceptable

Comments on Format (Required):

- CHOP comprehensive responsible conduct of research program: online module, live, in-person discussion and seminar

Comments on Subject Matter (Required):

- Authorship, publication, collaborative science, peer review, mentorship, data sharing and ownership, research misconduct, conflicts of interest, intellectual property

Comments on Faculty Participation (Required; not applicable for mid- and senior-career awards):

- Yes, faculty are involved with training

Comments on Duration (Required):

- 8 hours training, 4 hours live or in-person discussion, 2 hours seminar, and additional hours other readings etc.

Comments on Frequency (Required):

- Complete every 3 years

Resource Sharing Plans:

Acceptable

Authentication of Key Biological and/or Chemical Resources:

Not Applicable (No Relevant Resources)

Budget and Period of Support:

Recommend as Requested

CRITIQUE 2

Candidate: 1
Career Development Plan/Career Goals: 1
Research Plan: 2
Mentor(s), Co-Mentor(s), Consultant(s), Collaborator(s): 1
Environment Commitment to the Candidate: 1

Overall Impact: This is a new K23 application entitled "A Pilot Randomized Controlled Trial of Resilience Coaching for Adolescents with Chronic Musculoskeletal Pain." This application builds on preliminary studies developing a resilience-training intervention program for adolescents and young adults with serious illness called Promoting Resilience in Stress Management (PRISM). The current application proposes to assess impact on pain related clinical outcomes in a pilot randomized controlled trial with the goal to develop an R01 application for a multi-center trial. Pain is an area of high interest in pediatric and adolescent care and this proposal has potential for high impact on pain management practices. The exceptional resources and institutional support at Children's Hospital of Philadelphia and the University of Pennsylvania, outstanding multidisciplinary mentorship team, and proposed career development activities support enthusiasm that the candidate can achieve her long-term goal of becoming an independent investigator and expert in pediatric chronic pain, resilience, and behavioral health interventions for adolescents with CMP. There are minor weaknesses in the research plan that moderate enthusiasm, but overall, this proposal is solidly developed and is anticipated to serve well to advance knowledge in this area of high need and successfully advance the development of this applicant.

1. Candidate:
Strengths

- Dr. Gmuca is an Assistant Professor of Pediatrics and Director of the Center for Amplified Pain Syndromes at CHOP a nationally recognized center of excellence in Pain management. She has background in pediatric rheumatology and clinical research, with specific training in primary and secondary data analyses and mixed methods research (Master of Science in Clinical Epidemiology). Her long-term goal is to become a renowned clinician scientist with expertise in the treatment and management of chronic musculoskeletal pain
- Dr. Gmuca has a productive track record, including 5 1st author and 2 senior author papers directly related to proposed research within the past 3 years. She has successfully competed for funding and is currently supported by an RRF Investigator Award and a K-Bridge Award. She has received multiple early investigator recognition awards (ACR Distinguished Fellow, CHOP Distinguished Research Trainee, Thrasher Early Career Award). She is well-established and well-respected in local and national clinical research groups studying chronic pain

Weaknesses

- No significant weaknesses

2. Career Development Plan/Career Goals & Objectives:
Strengths

- The application presents a well-focused structured mentoring plan including a plan for didactic training and guided learning in advanced statistical analysis, clinical trials, mixed-methods research, implementation science, resilience, and pediatric chronic pain.
- There is a detailed plan describing short- and medium-term career goals including developing further skill in analytic methods and expertise in mixed-methods, intervention adaption and refinement and implementation science. There are adequate plans for monitoring and evaluating the candidate's research and career development progress the end goal of this project is development of an R01 application to fund a multi-center trial.

Weaknesses

- No significant weaknesses

3. Research Plan:
Strengths

- The applicant has performed focused and rigorous prior research supporting the proposal, including a prelim study published in *J Pediatr* demonstrating low levels of resilience as well as moderate associations between resilience and symptom severity in adolescent CMP. This result was extended with an RRF funded pilot single-arm feasibility and acceptability trial of a resilience coaching program, Promoting Resilience in Stress Management (PRISM), in 27 youth with CMP. This study demonstrated high feasibility and acceptability of PRISM delivery in this patient population. Participant feedback led to modification of the original program to incorporate a patient group session (PRISM-C)
- In this K23 proposal Dr. Gmuca proposes to conduct a pilot phase 2 randomized controlled trial of PRISM-C compared to usual care (UC) for 120 teens aged 12-17 years, testing the hypothesis that PRISM-C will lead to improved pain-related outcomes (functional disability, pain intensity, psychological distress) via theorized intervention targets (resilience, mindfulness, benefit-finding).
- The proposal has 3 aims: 1) Evaluate impact on pain-related clinical outcomes; 2) Explore potential patient- caregiver moderators (e.g., age, sex, anxiety, depression); 3) study implementation outcomes (feasibility, acceptability, appropriateness) and barriers and facilitators to engagement
- The scientific rationale for this proposal is well-supported by the preliminary evidence and the research strategies presented appear robust and well- designed to provide preliminary efficacy data and identify moderators, as well as barriers and facilitators to implementation, to inform a subsequent R01-funded multicenter RCT.
- The population size and statistical approach seem appropriate, and implementation should be feasible given the number of patients attending the CHOP Rheumatology clinic and chronic pain programs.

Weaknesses

- Assumptions regarding retention in the study may be generous- the applicant has outlined a number of mitigation strategies that improve enthusiasm
- Comparison to usual care (recommendation for CBT) is subject to operator bias depending on vigor of the recommendations. Increased uptake would diminish the likelihood of measurable impact of the PRISM-C program. Using this as the standard, however, is most relevant for consideration of future use in unstructured clinical care settings.

4. Mentor(s), Co-Mentor(s), Consultant(s), Collaborator(s):
Strengths

- The Scientific Advisory Committee is well-balanced and appropriate for this research proposal. There is a robust structured mentorship plan with an appropriate career development timeline
- Mentor- Pamela Weiss- Associate Professor with high achievement for stage of career. Multiple awards for research, multiple R01 funded clinician investigator, successful track record of

mentorship and training. She has had a long-term productive mentoring relationship with the candidate that supports the likelihood of success.

- Jami Young- Assoc. Professor Psychiatry- expertise in clinical psychology, clinical trials, and mental health-related interventions
- Tonya Palermo- Professor Pain Medicine and Psychiatry- expertise in pediatric chronic pain research and psychosocial interventions for pain management
- Abby Rosenberg- Assoc. Professor Hematology/ Oncology- expertise in development and rigorous testing of psychosocial interventions for children with serious illness and their families. Specific focus on the construct of resilience
- Peter Cronholm- Assoc. Professor, Clinical Epidemiology- expertise in the use of qualitative and mixed methods research methodologies with a focus on integrating key stakeholder perspectives and goals into research designs
- Rui Xiao- Assoc. Professor, Biostatistics- expertise in biostatistics and statistical genetics

Weaknesses

- No significant weaknesses

5. **Environment:**

Strengths

- CHOP is an exceptionally vigorous research environment and eminently suitable for support of this proposal.
- There is clear and strong evidence of institutional and individual mentor support for the applicant, including guaranteed protected time and access to the resources and expertise required for success.

Weaknesses

- No weaknesses

Study Timeline:

Strengths

- The study timeline is described in detail and seems appropriate. Dr. Gmuca and her advisors are experienced in conducting similar studies and have shown success. The enrollment targets appear feasible and implementation methods well-justified.
- The applicant has discussed potential challenges to accrual and analysis and provided appropriate mitigation strategies

Weaknesses

- None noted

Protections for Human Subjects:

Acceptable Risks and Adequate Protections

Data and Safety Monitoring Plan (Applicable for Clinical Trials Only):

Acceptable

Inclusion Plans:

- Sex/Gender: Distribution justified scientifically
- Race/Ethnicity: Distribution justified scientifically
- For NIH-Defined Phase III trials, Plans for valid design and analysis:
- Inclusion/Exclusion Based on Age: Distribution justified scientifically
- This study is specifically oriented to adolescents. Chronic pain sources and management strategies vary substantially based on stage of intellectual development, making it necessary to focus research with specified age-groups

Training in the Responsible Conduct of Research:
Acceptable

Comments on Format (Required):

- Didactic and independent study

Comments on Subject Matter (Required):

- Appropriate

Comments on Faculty Participation (Required; not applicable for mid- and senior-career awards):

- Appropriate

Comments on Duration (Required):

- Appropriate

Comments on Frequency (Required):

- Appropriate

Resource Sharing Plans:

Acceptable

Budget and Period of Support:

Recommend as Requested

CRITIQUE 3

Candidate: 1
Career Development Plan/Career Goals: 1
Research Plan: 3
Mentor(s), Co-Mentor(s), Consultant(s), Collaborator(s): 1
Environment Commitment to the Candidate: 1

Overall Impact: This proposal is from an emerging investigator with fellowship training in pediatric rheumatology and a master's degree in clinical epidemiology. The primary objective of the research

proposal and training program is to gain expertise in resilience, pediatric chronic musculoskeletal pain (CMP), and clinical trials. The training program is well designed to achieve her career goals. The strengths of the proposal include multiple committed mentors, weekly meetings and a structured evaluation plan, exposure to experts in all relevant fields, and the necessary course work to improve her knowledge in clinical trial design, clinical pain psychology, and implementation science. The research proposal seeks to complete a pilot RCT comparing the PRISM-C resilience program versus usual care in 120 youth with CMP. The outcomes of interest include reduction in functional disability, psychological distress, and pain; as well as the intervention targets such as resilience, mindfulness, and benefit-finding. The secondary objectives explore moderators of the PRISM-C program and semi-structured interviews to solicit barriers and facilitators of the program. The proposal and methods are well designed and thoughtful. Overall, these strengths provide significant enthusiasm for the project. However, it should be noted that there is some discordance between the study's background and its aims. The significance of the research strategy outlines the importance of the topic and the challenges of accessing CBT or resilience coaching. Similarly, the proposal highlights the use of PRISM in several other populations and provides preliminary data of its effectiveness in the target CMP population. As a result, it is unclear what is the most important knowledge gap to be addressed: "does PRISM work?", "Who does it work best for?", or "How can we make it work better?" Depending on the knowledge gap to be addressed, the research design would likely benefit from a more novel RCT design (randomize responders only, randomize PRISM vs PRISM-C, randomize the use of facilitators or not) or even a simple cohort design to generate a large sample to understand potential differences in responder's vs non-responders. Additionally, unlike the initial single-arm pilot study, the potential for recruitment challenges of a RCT that randomizes to usual care are not adequately addressed. Despite these minor weaknesses, this proposal will support the development of a strong emerging investigator and likely lead to an independent research career investigating interventions to reduce CMP.

THE FOLLOWING SECTIONS WERE PREPARED BY THE SCIENTIFIC REVIEW OFFICER TO SUMMARIZE THE OUTCOME OF DISCUSSIONS OF THE REVIEW COMMITTEE, OR REVIEWERS' WRITTEN CRITIQUES, ON THE FOLLOWING ISSUES:

PROTECTION OF HUMAN SUBJECTS: ACCEPTABLE

INCLUSION OF WOMEN PLAN: ACCEPTABLE

INCLUSION OF MINORITIES PLAN: ACCEPTABLE

INCLUSION ACROSS THE LIFESPAN: ACCEPTABLE

TRAINING IN THE RESPONSIBLE CONDUCT OF RESEARCH: ACCEPTABLE

COMMITTEE BUDGET RECOMMENDATIONS: The budget was recommended as requested.

Footnotes for 1 K23 AR081409-01; PI Name: GMUCA, SABRINA ANDREA

NIH has modified its policy regarding the receipt of resubmissions (amended applications).See Guide Notice NOT-OD-18-197 at https://grants.nih.gov/grants/guide/notice-files/NOT-OD-18-197.html. The impact/priority score is calculated after discussion of an application by averaging the overall scores (1-9) given by all voting reviewers on the committee and

multiplying by 10. The criterion scores are submitted prior to the meeting by the individual reviewers assigned to an application, and are not discussed specifically at the review meeting or calculated into the overall impact score. Some applications also receive a percentile ranking. For details on the review process, see http://grants.nih.gov/grants/peer_review_process.htm#scoring.
